# The interplay of aggression and psychopathy in a correctional treatment setting

**DOI:** 10.1192/j.eurpsy.2024.1203

**Published:** 2024-08-27

**Authors:** A. Voulgaris, P. Briken, E. Stück

**Affiliations:** ^1^Institute for Sex Research, Sexual Medicine and Forensic Psychiatry, Medical University Hamburg, Hamburg, Germany

## Abstract

**Introduction:**

Aggression is a relevant risk factor for criminal behavior. Psychopathy is known to correlate with a higher risk for violent offenses and research suggests that successful therapy of psychopathy is complicated.

**Objectives:**

Our goal was to explore the overlap between psychopathy and aggression and the specific influence of psychopathic traits on change in aggression during correctional therapy.

**Methods:**

A pre-post-study rating psychopathy and aggression in men imprisoned for sexual and non-sexual violent offenses aged between 20 and 67 (M=37.6, SD 11.6) was conducted. The participants filled out standardized pre- and post-treatment ratings after admission and after an average of 16 months (n=144 for pre-rating, n=89 for post-rating). Psychopathy was measured via the PCL-R and aggression with the BDHI (Buss-Durkee Hostility Inventory).

We calculated two-tailed Pearson correlations for BDHI Pre-, Post-, and Change Scores and the PCL-R. Further, the BDHI pre-post-differences were compared using independent t-Tests, effect sizes were calculated using Cohen’s d (small, medium, and large effect sizes are d = .20, .50, and .80). Also, unpaired t-tests were carried out to compare between participants with lower and higher PCL-R sum scores (median split, mdn= 16.8, M=16.8, SD=7.0).

**Results:**

Psychopathy facets 3 and 4 (lifestyle, antisocial) and the sum score correlate significantly with the pre-, and post-BDHI total score and the subscale direct hostility but not with indirect hostility. Regarding BDHI change scores, only the interpersonal facet of PCL-R correlated significantly with direct hostility and the total BDHI score. In the whole population, a significant reduction of the BDHI was only found in the subscale indirect hostility (p=.015, cohens d= .26). In the subgroup of individuals with lower PCL-R (<16.8) showed a reduction of indirect hostility (p<.001, cohens d= .50) and the total BDHI score (p=.003, cohens d=.42). Interestingly, in the group with higher PCL-R scores no significant reduction of self-assessed hostility via BDHI was observable during therapy.

**Conclusions:**

We identified a significant correlation between psychopathy and aggression, especially regarding facets three, four, and the sum score. Only the interpersonal facet correlated with the change in aggression during treatment in prison. In the group with higher psychopathic traits, no change in aggression was achievable during therapy. Thus, in the aspect of aggression and hostility, our data suggest that higher psychopathic traits may be viewed as a complicating factor for successful therapy.

**Disclosure of Interest:**

None Declared

